# IB4(+) and TRPV1(+) sensory neurons mediate pain but not proliferation in a mouse model of squamous cell carcinoma

**DOI:** 10.1186/1744-9081-10-5

**Published:** 2014-02-13

**Authors:** Yi Ye, Sam S Bae, Chi T Viet, Scott Troob, Daniel Bernabé, Brian L Schmidt

**Affiliations:** 1 Bluestone Center for Clinical Research, New York University, New York, USA; 2 Department of Oral and Maxillofacial Surgery, University of Michigan, Ann Arbor, USA; 3 Department of Oral Maxillofacial Surgery, New York University, New York, USA; 4 Department of Otolaryngology, Head and Neck Surgery, New York University, New York, USA; 5 Oral Oncology Center, Aracatuba Dental School, University of Estadual Paulista, Aracatuba, San Paulo, Brazil

**Keywords:** Isolectin B4, TRPV1, Squamous cell carcinoma, Cancer pain, Proliferation

## Abstract

**Background:**

Cancer pain severely limits function and significantly reduces quality of life. Subtypes of sensory neurons involved in cancer pain and proliferation are not clear.

**Methods:**

We produced a cancer model by inoculating human oral squamous cell carcinoma (SCC) cells into the hind paw of athymic mice. We quantified mechanical and thermal nociception using the paw withdrawal assays. Neurotoxins isolectin B4-saporin (IB4-SAP), or capsaicin was injected intrathecally to selectively ablate IB4(+) neurons or TRPV1(+) neurons, respectively. JNJ-17203212, a TRPV1 antagonist, was also injected intrathecally. TRPV1 protein expression in the spinal cord was quantified with western blot. Paw volume was measured by a plethysmometer and was used as an index for tumor size. Ki-67 immunostaining in mouse paw sections was performed to evaluate cancer proliferation *in situ*.

**Results:**

We showed that mice with SCC exhibited both mechanical and thermal hypersensitivity. Selective ablation of IB4(+) neurons by IB4-SAP decreased mechanical allodynia in mice with SCC. Selective ablation of TRPV1(+) neurons by intrathecal capsaicin injection, or TRPV1 antagonism by JNJ-17203212 in the IB4-SAP treated mice completely reversed SCC-induced thermal hyperalgesia, without affecting mechanical allodynia. Furthermore, TRPV1 protein expression was increased in the spinal cord of SCC mice compared to normal mice. Neither removal of IB4(+) or TRPV1(+) neurons affected SCC proliferation.

**Conclusions:**

We show in a mouse model that IB4(+) neurons play an important role in cancer-induced mechanical allodynia, while TRPV1 mediates cancer-induced thermal hyperalgesia. Characterization of the sensory fiber subtypes responsible for cancer pain could lead to the development of targeted therapeutics.

## Background

Cancer patients in pain suffer with a poor quality of life. Approximately 75% to 90% of patients with advanced cancer have pain that is particularly difficult to treat as there is no effective analgesic available in treating intractable cancer pain [1,2].

One strategic approach to cancer pain drug development is targeted therapy against subtypes of nociceptors that control specific sensory modalities. C-nociceptors, are a heterogeneous population of neurons that are broadly divided into two subclasses. The peptidergic class does not bind isolectin B4 (IB4), but expresses TRPV1, nerve growth factor (NGF) receptor TrkA, and neuropeptides such as calcitonin gene-related peptide (CGRP) and substance P (SP) [3,4]. The second subtype is nonpeptidergic neurons, which binds IB4, expresses glial cell-derived neurotrophic factor (GDNF) receptors and P2X3 receptors, and has poor expression of SP and CGRP [5-8]. In the spinal cord, IB4(+) neurons terminate predominantly in inner lamina II whereas IB4(-) neurons terminate in lamina I and outer lamina (II) [3,9]. The distinct neurochemical and anatomical characteristics of the two subsets lead some researchers to hypothesize that they have different functional properties in conveying nociceptive information [10-14]. Recently, it has been proposed that nonpeptidergic IB4(+) neurons and the peptidergic TRPV1(+) neurons represent two parallel neuronal pathways that selectively control mechanical and thermal pain, respectively [10,12]. However, many other studies did not find complete segregation between the IB4(+) and TRPV1(+) neurons in mechanical vs. thermal pain [15-18]. Some authors also hypothesized that the two neuronal populations serve different functions in pathological pain conditions with IB4(+) neurons specifically contributing to neuropathic pain while the TRPV1(+) neurons contribute to inflammatory pain [19,20].

The differential involvement of IB4(+) and TRPV1(+) neurons in cancer pain has not been well-characterized. Our previous studies have demonstrated that the SCC microenvironment contains high levels of NGF, which mediates both mechanical and thermal nociception in mouse models of SCC [21,22]. NGF is known to upregulate and sensitize TRPV1 [23-26]. Indeed, TRPV1 expression is upregulated in neurons of mouse and rat models of cancer [18,27,28]. TRPV1 also has been shown to mediate both mechanical and thermal nociception in a rat model of SCC [28]. In our mouse SCC supernatant model (i.e. injection of SCC supernatant into the hind paw of a naïve animal), mechanical allodynia is mediated by IB4(+) and NGF-responding neurons, whereas thermal hyperalgesia is mediated exclusively by NGF-responding neurons [22]. While the supernatant model is useful for studying the effect of secretory product from cancer cells on putative nociceptors, the effect of supernatant is short-lasting and does not fully mimic the cancer microenvironment. The specific roles of IB4(+) and TRPV1(+) neurons in the cancer microenvironment and their contribution to cancer pain need to be determined.

In addition to their role in cancer pain, neurons could also promote cancer progression [29,30]. Perineural invasion, a process by which cancer cells invade and proliferate within the nerve, is seen frequently in oral SCC [31-33]. Neurotransmitters released by the autonomic nervous system, modulate proliferation, apoptosis, angiogenesis, and metastasis [29]. Both surgical and chemical sympathectomy can suppress tumor growth and invasiveness [34,35]. A recent study shows that SP(+) neurons promote cancer proliferation and migration [36], suggesting that sensory neurons could also contribute to the process of cancer development.

Based on the above studies, we hypothesize that nonpeptidergic IB4(+) and peptidergic TRPV1(+) neurons play differential roles in cancer pain and proliferation. To test this hypothesis, we employ specific neurotoxins to ablate, and pharmacological agents to selectively antagonize, IB4(+) and TRPV1(+) neurons.

## Methods

### Cell culture

The human oral SCC cell line, HSC-3 (ATCC, Manassas, VA) was cultivated in Dulbecco’s Modification of Eagle’s Medium (DMEM) with 4.5 g/L glucose, l-glutamine and sodium pyruvate, supplemented with 10% fetal bovine serum, 25 μg/mL fungizone, 100 μg/mL streptomycin sulfate, and 100 U/mL penicillin G.

### Experimental animals

34 female athymic, immunocompromised BALB/c mice (6–8 weeks old, Charles River Laboratories, Hollister, CA) were used in this study. Mice were exposed to a 12 hour light–dark cycle and kept in a temperature-controlled room with food and water *ad libitum*. All procedures were approved by the New York University Institutional Animal Care and Use Committee.

### Intrathecal administration of drugs

Intrathecal injection of either IB4-SAP or capsaicin destroys central terminals of IB4(+) and TRPV1(+) neurons in the spinal cord and eventually kills the cell bodies of these two populations in the DRGs as previously reported [10,22,37-40]. We observed more than 50% reduction in IB4 immunopositivity in the spinal cord 2 weeks after IB4-SAP treatment [22]. All mice received intrathecal injection of drugs while anesthetized with 2.5% isoflurane. Drugs were delivered into the subarachnoid space between the L4 and L5 vertebrae in the spinal cord via a 10 μl Hamilton syringe. 1.53 mM IB4-saporin (IB4-SAP, 53% saporin/mole IB4) or 0.81 mM saporin (SAP, Advanced Targeting Systems, San Diego, CA) was diluted with PBS to a total volume of 8 μl, and were injected 2 weeks before SCC cell inoculation; as SAP has no method of cell entry on its own and does not change thermal or mechanical behaviours compared to naive mice [22], it was used as a control. Capsaicin (4 mM, Sigma Aldrich, St. Louis, MO), or vehicle (10% ethanol, 10% Tween 80, 80% saline) at a volume of 8.0 μl was injected 3 weeks post SCC inoculation (day 19). The TRPV1 antagonist JNJ-17203212 (30 μM in 8 μl 5% DMSO and 95% PBS, Sigma Aldrich, St. Louis, MO), or vehicle was injected intrathecally at week 5 after SCC inoculation.

### Mouse cancer model

A paw SCC mouse model was used because paw withdrawal is the gold standard for quantifying mechanical and thermal behaviors, and because of technical challenges with the behavioral measurement and tumor size monitoring in the oral region [21]. After recording baseline behavior measurements, mice were injected with 10^6^ HSC-3 cells in a 50 μl vehicle consisting of a mixture of DMEM and Matrigel™ (Becton Dickinson & Co., Franklin Lakes, NJ) into the plantar surface of the right hind paw.

### Paw volume measurement

Paw volume measurement was performed with a plethysmometer (IITC Life Sciences, Woodland Hills, CA) and taken as an index for tumor growth. Triplicate measurements were taken for each time point.

### Behavioral testing

Mechanical allodynia was quantified using an electronic von Frey anesthesiometer (IITC Life Sciences, Woodland Hills, CA). The withdrawal threshold was defined as the force (g) sufficient to elicit a withdrawal response. Six measurements were taken for each animal and the mean was used as threshold for each animal at each time point. Thermal hyperalgesia was measured using a paw thermal stimulator (IITC Life Sciences, Woodland Hills, CA). Mice were placed in a plastic chamber on a heated glass surface (25ºC). A radiant heat source was focused on the hind paw and latency to withdraw was measured as the average of 6 trials per animal taken ≥ 5 minutes apart. The cut-off latency was set at 20 seconds to avoid tissue damage. Behavioral tests were performed at 2 weeks following IB4-SAP injection right before SCC inoculation (baseline), at post inoculation week 2 (day 11) and week 4 (day 25). At post inoculation week 5 (day 32), the behavioral measurement was taken 30 min after drug administration. The observer was blinded to treatment groups in all behavioral experiments. The mean of mechanical thresholds or thermal latency for each group of animals was compared at each individual time point. No group differences were found at baseline for both mechanical and thermal assays.

### Euthanasia and tissue processing

Mice were euthanized with isoflurane and perfused transcardially with 0.1 M phosphate buffer saline (PBS) at week 5 following SCC inoculation. The lumbar section of the spinal cord was dissected, snap-frozen in liquid nitrogen and stored at -80ºC. Hind paws were removed, post-fixed in 4% formaldehyde, and cryoprotected in sucrose gradient (20%-50%) at 4ºC. Serial frozen paw sections (20 μm) were cut with a cryostat and thaw-mounted on gelatin-coated slides for processing.

### Western blotting

The spinal cord was lysed in ice-cold RIPA buffer with a protease inhibitor cocktail (Sigma Aldrich, St. Louis, MO). Tissues were homogenized using a tissue homogenizer. Spinal cord samples were loaded into the 10% polyacrylamide gels and then were electrotransferred onto a nitrocellulose membrane. The membranes were blocked with 10% non-fat dry milk in PBS with 0.3% Tween for 1 hour and then incubated overnight at 4°C with rabbit anti-TRPV1 antibody (1:500; Alomone Labs, Jerusalem, Israel), or GAPDH antibody (1:5000; Sigma Aldrich, St. Louis, MO) diluted in PBS. The membrane was then incubated in horseradish peroxidase-conjugated anti-rabbit IgG (1:5000; Santa Cruz Biotechnology, Dallas, TX) for 2 hours at room temperature. The antigen–antibody complexes were visualized using an enhanced chemiluminescence detection reagent (Bio-Rad Laboratories, Hercules, CA). Bands were scanned using a densitometer (GS-700; Bio-Rad Laboratories, Hercules, CA), and quantification was performed using NIH Image J software.

### Ki-67 labeling

Paw sections were stained for Ki-67 (rabbit anti-Ki67, 1:500; Abcam, Cambridge, MA), a nuclear protein employed to quantify proliferation. After incubation in the primary antibody, sections were rinsed in PBS three times for 10 min each, and then incubated in the Texas Red goat anti-rabbit secondary antibody (1:700; Jackson ImmunoResearch Laboratories, West Grove, PA) for 1 hour at room temperature. Image analysis was performed using NIH Image J software. The area of staining was outlined and pixel density within the selected area was then measured and divided by the total area. Data were collected from 5 randomly selected sections from a minimum of 5 animals per treatment group.

### Statistical analysis

The statistics software SigmaPlot for Windows (version 11.0) was used to perform the statistical analysis. One-way ANOVA with a Tukey Multiple Comparisons post-hoc test was used to compare the experimental groups. Significance level was set at p < 0.05. Results were presented as mean ± standard error (SE).

## Results

### SCC induced both mechanical allodynia and thermal hyperalgesia

**Figure 1:**
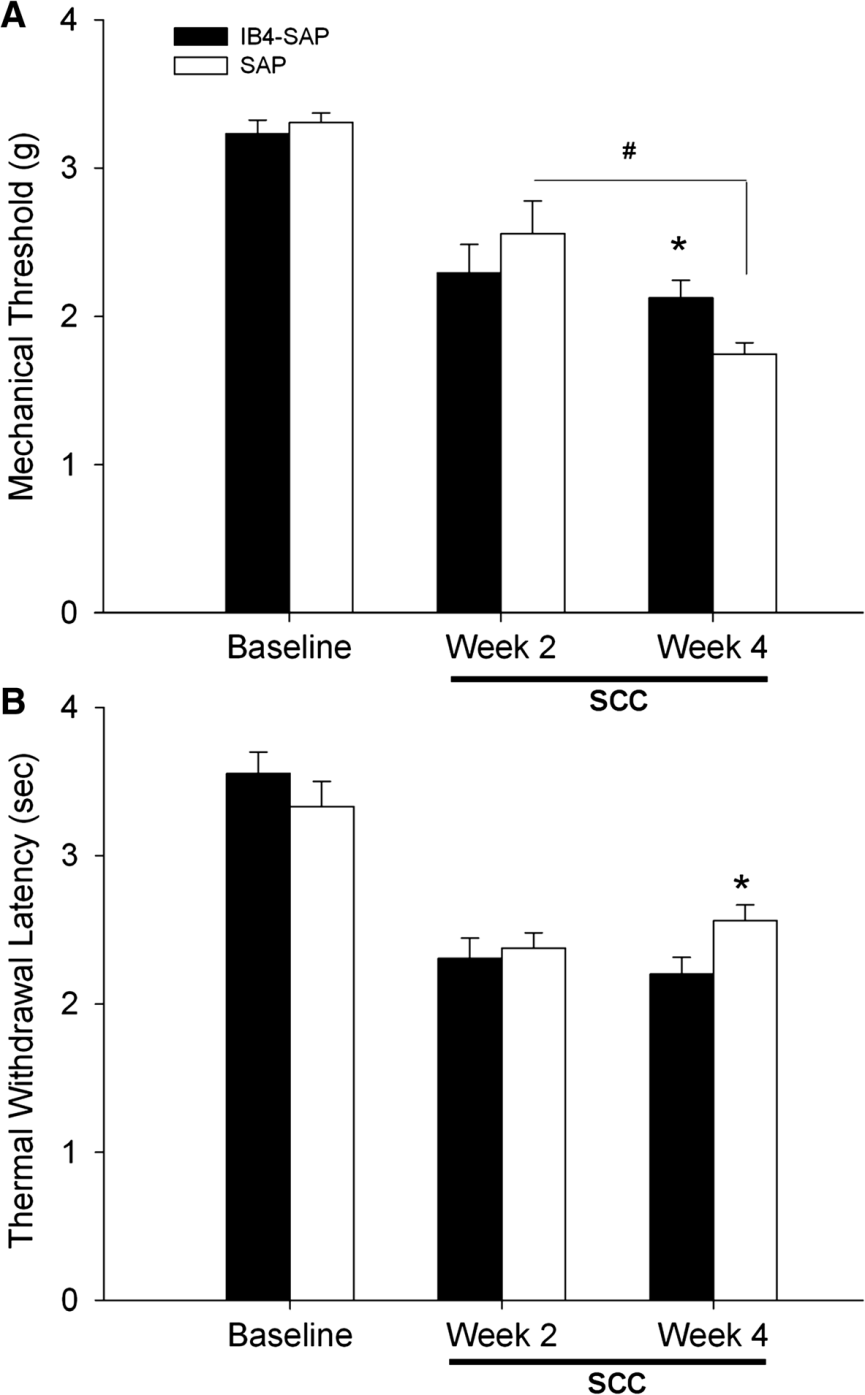
**Nociceptive behavior in IB4-SAP and SAP treated SCC mice. A**. Mechanical thresholds were decreased from baseline following SCC inoculation in both treatment groups (P < 0.001 for baseline vs. week 2 and week 4 in the IB4-SAP and SAP groups). IB4-SAP (N = 8) and SAP (N = 8) treated mice had similar mechanical thresholds at baseline and at post inoculation week 2. However, IB4-SAP significantly reduced SCC-induced mechanical allodynia compared to SAP treated mice at week 4 post inoculation (*, P < 0.05). While no difference in mechanical thresholds was found in IB4-SAP group between week 2 and week 4, mechanical thresholds were significantly reduced in SAP mice from week 2 to week 4 (#, P < 0.001). **B**. Thermal latency was decreased from baseline following SCC inoculation (P < 0.001 for baseline vs. week 2 and week 4 in the IB4-SAP and SAP groups). IB4-SAP and SAP treated mice had similar thermal latency at baseline and at week 2 post inoculation. IB4-SAP treatment significantly increased thermal hyperalgesia compared to SAP treated mice at week 4 post inoculation (*, P < 0.05).

2 weeks following intrathecal injection, IB4-SAP treated mice exhibited similar baseline mechanical or thermal behaviour compared to SAP treated mice (Figure 1A). After SCC inoculation, both SAP and IB4-SAP treated mice exhibited decreased paw withdrawal thresholds to mechanical stimulation compared to their pre-inoculation thresholds throughout the four week observation period (Figure 1A). Mice with SCC also demonstrated decreased thermal latency post-inoculation compared to their pre-inoculation baseline (Figure 1B).

### IB4-SAP treatment reduced mechanical allodynia, but increased thermal hyperalgesia

While both vehicle control mice and IB4-SAP treated mice developed mechanical allodynia following SCC inoculation observed at post-inoculation week 2 to week 4, IB4-SAP treatment led to significant attenuation of mechanical allodynia at week 4, but not at week 2, compared to mice (Figure 1A). However, IB4-SAP treatment led to increased thermal hyperalgesia at week 4 post SCC inoculation, compared to the control SAP-treated group (Figure 1B).

### SCC induced thermal hyperalgesia was mediated by TRPV1

**Figure 2:**
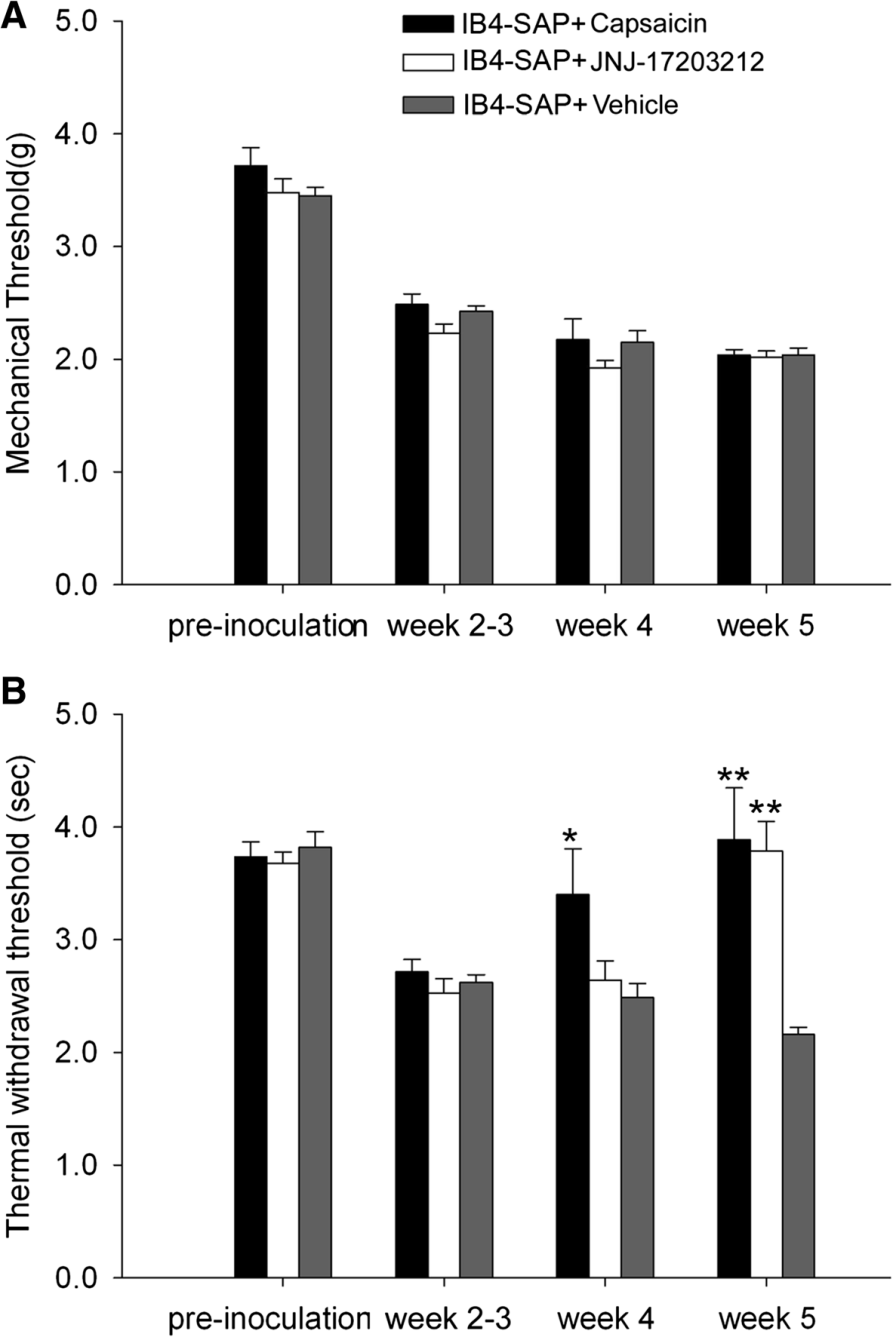
**Nociceptive behavior in IB4-SAP treated SCC mice injected with capsaicin and the TRPV1 antagonist (JNJ-17203212).** Six mice were used in each group. All mice were treated with IB4-SAP 2 weeks before SCC inoculation. Capsaicin was injected intrathecally at week 3 post inoculation. JNJ-17203212 was injected intrathecally 30 min before the behavior measurement at week 5. **A**. No difference in mechanical thresholds was found among capsaicin, JNJ-17203212, or vehicle injected SCC mice throughout the observation time (5 weeks after SCC inoculation). All animals in the capsaicin, JNJ-17203212, and vehicle injected groups had reduced mechanical thresholds from baseline at week 2, week 4, and week 5 relative to baseline (P < 0.001 in all measurements). **B**. Capsaicin injection significantly reversed thermal hyperalgesia at weeks 4 and 5, one and two weeks after capsaicin injection, respectively (*, P < 0.05; capsaicin vs. vehicle or JNJ-17203212 at week 4; **, P < 0.01 capsaicin vs. vehicle at week 5). JNJ-17203212 injection also reversed thermal hyperalgesia at week 5 (**, P < 0.01, JNJ-17203212 vs. vehicle).

**Figure 3:**
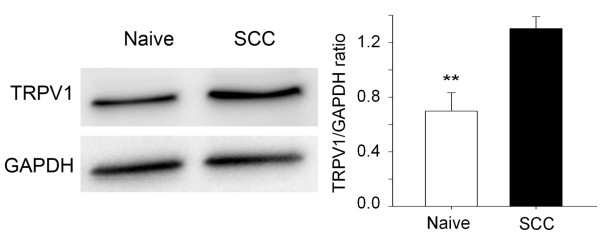
**Spinal TRPV1 protein was increased in cancer mice.** TRPV1 protein expression was increased in the spinal cords of SCC mice (N = 6) at week 5 following SCC inoculation compared to naive mice (N = 5). Western blots were performed to isolate and measure TRPV1; GAPDH was used as the reference protein. **, P < 0.01.

To determine whether TRPV1 contributed to thermal hyperalgesia induced by SCC inoculation and IB4-SAP treatment, we quantified nociceptive behavior after intrathecal injection of capsaicin into IB4-SAP pre-treated mice during post SCC inoculation week 3. Capsaicin, a neurotoxin that destroys central terminals of TRPV1(+) neurons [10], significantly reduced SCC-induced thermal hyperalgesia without affecting mechanical allodynia, at post SCC inoculation week 4 and 5 (i.e., one week and two weeks after capsaicin treatment) (Figure 2). Similarly, TRPV1 antagonism by intrathecal JNJ-17203212 at week 5 in IB4-SAP treated mice with SCC also reversed thermal hyperalgesia with no effect on mechanical allodynia (Figure 2). To determine whether the thermal hyperalgesic effect was accompanied by increased expression of TRPV1 we measured TPRV1 protein levels in the spinal cord. Western blotting showed that TRPV1 expression was increased in the spinal cord of mice with SCC compared to untreated, normal (naïve) mice (Figure 3). TRPV1 protein expression was increased in mice with SCC that were treated with IB4-SAP versus those that were treated with control SAP, although this trend was not statistically significant (data not shown). The thermal hyperalgesic effect that was induced by SCC inoculation and exacerbated by IB4-SAP treatment, was therefore mediated by TRPV1.

### IB4(+) and TRPV1(+) neurons did not affect SCC proliferation

**Figure 4:**
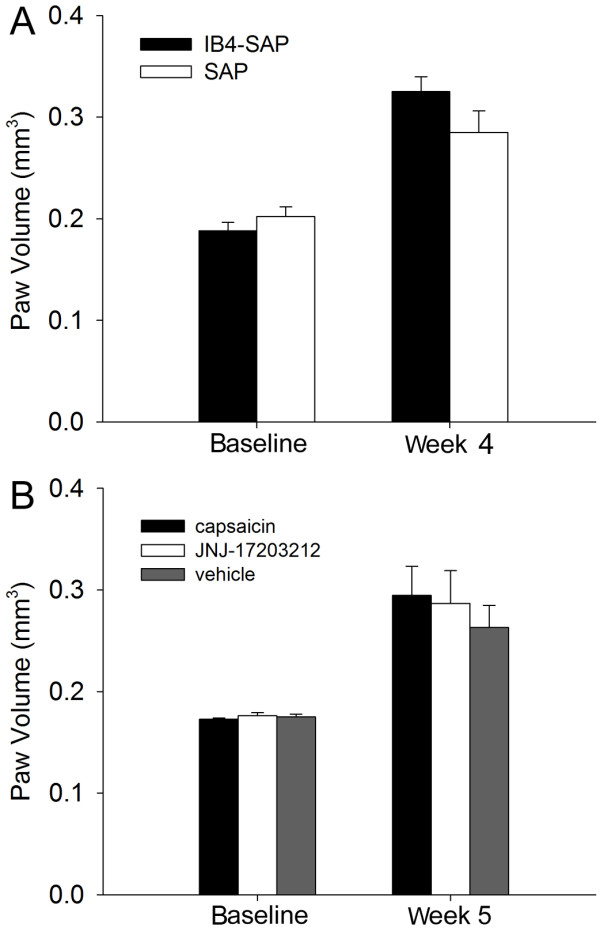
**Paw volume was not different across SCC mice treatment groups. A**. Paw volume was not different between SAP and IB4-SAP treated SCC mice (N = 6 in each group) at baseline or at week 4. **B**. Paw volume was not different among capsaicin, JNJ-17203212, or vehicle injected SCC mice at baseline or at week 5. *, P < 0.05; **, P < 0.01.

**Figure 5:**
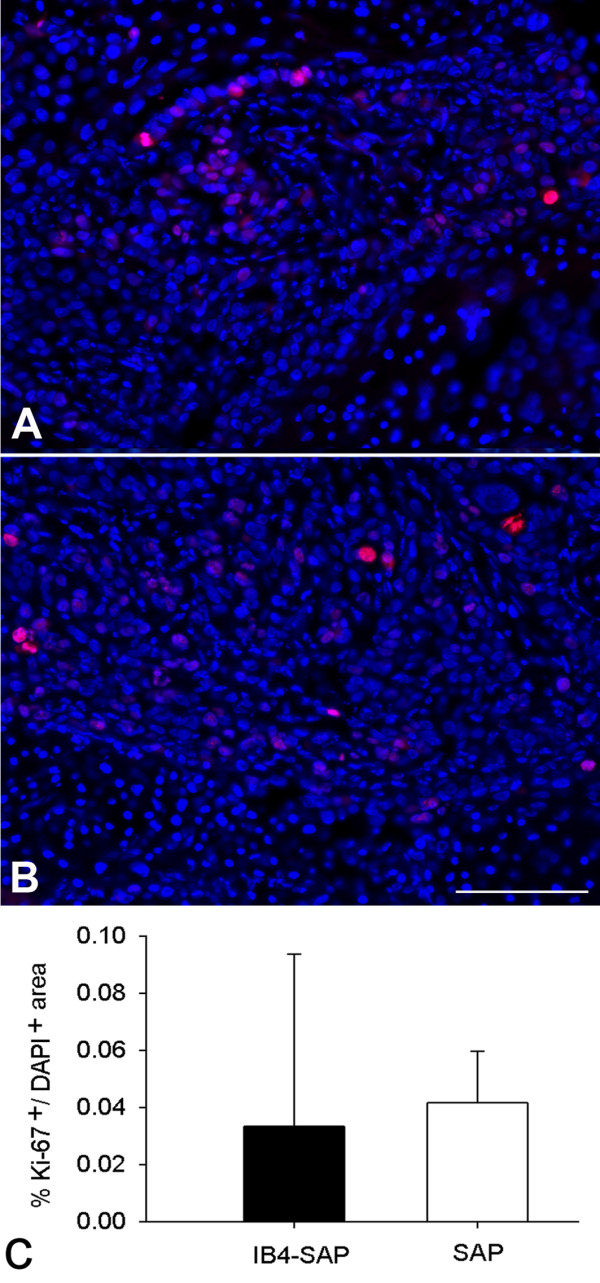
**Ki-67 and 4′,6-diamidino-2-phenylindole (DAPI) immunostaining in paw sections processed at weeks 5 following SCC inoculation. A**. An example of Ki-67 and DAPI staining in the paw section of IB4-SAP treated SCC mouse. **B**. An example of Ki-67 and DAPI staining in the paw section of SAP treated SCC mouse. **C**. Quantification of Ki-67-positive cells in total DAPI-positive cells showed no difference between IB4-SAP and SAP treated mice. Horizontal scale bar, 100 μm.

We found that selective ablation of IB4(+) neurons with IB4-SAP treatment only affected SCC-induced nociception (i.e. decreased mechanical allodynia and increased thermal hyperalgesia compared to control), but not SCC proliferation. Paw volume measurements were not significantly different between mice with SCC treated with IB4-SAP and those treated with control SAP (Figure 4A); paw volume measurements were comparable to our previous report in mice inoculated with SCC [21]. Ablation of TRPV1(+) neurons also had no further effect on paw tumor volume in mice with SCC treated with IB4-SAP (Figure 4B). In histological sections of paw SCC, both IB4-SAP and SAP-treated mice had similar proportions of Ki-67-positive cells among DAPI-positive cells (Figure 5).

## Discussion

The cancer microenvironment is densely innervated; however, the specific sensory fiber types responsible for cancer pain are not known. Behavioral characterization of the peripheral neuronal subtypes responsible for cancer pain would lend itself to targeted pharmacologic management of cancer pain. Here, using a cancer pain mouse model we selectively ablated two separate populations of putative nociceptors innervating the cancer microenvironment and observed distinct behavioral changes. Selective ablation of each of these fiber types did not impact cancer proliferation.

Our data suggest that IB4(+) and TRPV1(+) neurons have functionally distinct roles in cancer pain, at least in the level of mouse DRG and spinal cord, where few IB4(+) neurons overlap with TRPV1 [10,41], in contrast to rat DRG [14,42]. Scherrer et al., [10] show that IB4(+) and TRPV1(+) neurons exclusively express delta-opioid receptors (DOR) and mu-opioid receptors (MOR), respectively. In these mice intrathecal DOR agonists reduce mechanical allodynia, while MOR agonists reduce thermal hyperalgesia [10]. Likewise, genetic ablation of IB4(+) neurons reduces mechanical hypersensitivity, but not thermal hypersensitivity. On the other hand, pharmaxcological ablation of TRPV1(+) neurons selectively abolishes thermal hypersensitivity without affecting mechanical hypersensitivity [12]. It should be noted, however, that there are studies suggesting that MOR and DOR are colocalized and do not mediate distinct pain behaviors [17,18,43]. In rats, IB4-SAP treatment affects both mechanical and thermal sensitivity [37,44], probably due to expression of TRPV1 on IB4(+) neurons in rats [14,42]. TRPV1 also has been shown to mediate both mechanical and thermal nociception in cancer models of rats and dogs [28,45,46]. Such differences in IB4(+) and TRPV1(+) function may due to differences in species, experimental approaches, disease models, and behavioral assays. Therefore, additional studies are needed to determine whether our results are restricted to our specific cancer model and strain of mice. Moreover, we used athymic mice which lack cell mediated immunity. It is unknown whether nociceptive fibers differ in either function or neurochemical expression in athymic mice compared to normal mice.

Nevertheless, the role of IB4(+) neurons in mechanical hypersensitivity is demonstrated by a large body of evidence [5,22,39,44,47-49]. Using intrasciatic IB4-SAP injection, IB4(+) neurons have been shown to mediate mechanical sensitivity in normal conditions and after injury [37,44]. In contrast to peripheral IB4-SAP treatment, intrathecal IB4-SAP treatment did not seem to cause behaviour changes at the basal conditions in our model. Basal mechanical hypoalgesia has not been shown in other studies using intrathecal IB4-SAP treatment in rats [39,40,47,48]. However, there is a clear evidence that IB4-SAP treatment reduces mechanical allodynia in animal models of neuropathic pain [39,44,47,48]. The role of IB4(+) neurons in cancer induced mechanical allodynia is just emerging. Mechanical allodynia is a cardinal, and often the initial, symptom in cancer patients [50-52]. Using a cancer pain mouse model that is produced by injecting SCC supernatant into the hind paw, we previously showed that extracellular secretions from the cancer, not growth of the cancer, directly produced mechanical allodynia [22]. SCC secretes neurturin, a neurotrophic factor of the GDNF family, which could activate and sensitize IB4(+) neurons to mechanical stimulation [22]. Mechanical transducers such as TRPA1 and P2X3, which are expressed on IB4(+) neurons, could also contribute to IB4(+) mediated mechanical pain [6,41,53,54].

Ablation of IB4(+) neurons only partially reduced SCC-induced mechanical allodynia. This partial antinociceptive effect might be a result of incomplete destruction of IB4(+) neurons by IB4-SAP treatment at the time of the nociceptive behavioral measurements. Previous studies have reported that 55-100% of IB4(+) neurons are abolished by 21 days following IB4-SAP treatment [37,44,53,55]. Using the same approach as we did here, a more than 50% decrease in IB4 immunointensity was observed 2 weeks following IB4-SAP treatment in C57BL/6 mice with no cancer [22]. In our study, cancer-induced mechanical allodynia was partially reduced, but not completely abolished, on day 46 post IB4-SAP treatment; it is likely that IB4(+) fiber destruction is not complete in our cancer model. Indeed, our preliminary experiments using immunofluorescent staining showed a 68% reduction of IB4(+) neurons in the DRGs of IB4-SAP treated mice compared to SAP-treated mice; similarly, a partial reduction of IB4(+) fibers in the spinal cord of IB4-SAP treated mice is also observed (data not shown). In addition, a separate subtype of neurons (i.e. IB4(-)/NGF-responsive neurons) could contribute to the residual mechanical allodynia observed in our IB4-SAP treated cancer mice. We have previously showed that IB4(-)/NGF-responsive neurons mediated SCC supernatant induced mechanical allodynia [21,22]. This IB4(-)/NGF fiber population either does not express TRPV1 or TRPV1 does not mediate mechanical stimulation in these fibers, as we did not observe a direct involvement of TRPV1 in SCC-induced mechanical hypersensitivity.

The observed thermal hyperalgesia in mice with SCC could result from SCC-mediated secretion of algogenic agents such as NGF, ATP, and endothelin-1, which are known to cause thermal hyperalgesia [21,56], possibly through the TRPV1 receptor [57-61]. TRPV1-dependent thermal hyperalgesia is present in animal models of SCC [22,27,28,61] and bone cancer [62,63]. TRPV1 antagonism reduces cancer-induced thermal hyperalgesia in these models [62]. We also demonstrated the role of TRPV1 in SCC-induced thermal hyperalgesia in our model. Unexpectedly, IB4-SAP treatment enhanced thermal hyperalgesia in mice with SCC. This enhanced thermal hyperalgesic effect was not present until four weeks after IB4-SAP treatment, and was completely abolished with TRPV1 antagonism. This enhanced thermal hyperalgesia observed in IB4-SAP treated mice at post SCC inoculation week 4 could result from compensatory TRPV1(+) neuronal sprouting secondary to the loss of IB4(+) neurons in the spinal cord. Evidence supporting neuronal sprouting following IB4-SAP treatment has been equivocal. In rats there is no increase in sprouting of peptidergic IB4(-) neurons in dorsal root ganglion (DRG) or spinal cord following ablation of nonpeptidergic IB4(+) neurons with IB4-SAP treatment during a 30-day observation period [64]. On the other hand, a separate study in rats shows that IB4-SAP ablation of IB4(+) neurons in the mental nerve results in significant sprouting of parasympathetic fibers into the upper dermis at week 3 following IB4-SAP treatment [55].

Compensatory sprouting of nerve fibers might explain why we did not observe an antiproliferative effect by ablation of either IB4(+) or both IB4(+) and TRPV1(+) neurons. Interaction between cancer cells and the nervous system leads to a reciprocal proliferative effect [30]. Cancer cells, including prostate and pancreatic, can stimulate neurite outgrowth [65]. The nervous system especially the sympathetic nervous system contributes to cancer proliferation, migration, invasion and metastasis [29,34,35,66]. Our finding that pharmacologic ablation of IB4(+) and TRPV1(+) subtypes did not lead to a decrease in proliferation could be due to compensatory sprouting of autonomic or sensory neurons which maintains cancer proliferation. It has to be noted, however, that capsaicin or TRPV1 antagonists applied directly to oral SCC can exert an anti-proliferative effect by a mechanism that is independent of TRPV1 [67]. Another explanation for our findings is that our chemical ablation methods (e.g. IB4-SAP or capsaicin) only destroy central terminals of DRG neurons but leaves peripheral terminals in the vicinity of the tumor relatively intact and functional. Therefore, the role of efferent neuronal activity in cancer proliferation remains inconclusive.

## Conclusions

Our study demonstrated distinct roles of nonpeptidergic IB4(+) and peptidergic TRPV1(+) neurons in mediating cancer-induced nociception. We determined that TRPV1(+) neurons are involved exclusively in cancer-induced thermal hyperalgesia, but not mechanical allodynia in our mouse paw SCC model. Identification of subpopulation of neurons mediating SCC-induced pain is of clinical importance as mechanical pain is a primary symptom of oral SCC patients. Drug therapy targeting specific nociceptors could lead to more effective treatment of cancer-induced mechanical pain.

## Competing interests

The authors declare that they have no competing interests.

## Authors’ contributions

YY designed the study and YY wrote the draft of the manuscript. SB performed behaviour assessments for the animals. YY inoculated cancer cells, performed drug injections, western blot, and immunostaining. DB and ST quantified immunostaining images. CTV helped edit the manuscript. BLS supervised the project and the writing of the paper. All authors contributed to and have approved the final manuscript.

## References

[B1] Meuser T Pietruck C Radbruch L Stute P Lehmann KA Grond S Symptoms during cancer pain treatment following WHO-guidelines: a longitudinal follow-up study of symptom prevalence, severity and etiology Pain 2001 10 247 257 10.1016/S0304-3959(01)00324-4 11514084

[B2] Schwei MJ Honore P Rogers SD Salak-Johnson JL Finke MP Ramnaraine ML Clohisy DR Mantyh PW Neurochemical and cellular reorganization of the spinal cord in a murine model of bone cancer pain J Neurosci 1999 10 10886 10897 10594070 10.1523/JNEUROSCI.19-24-10886.1999 PMC6784931

[B3] Molliver DC Radeke MJ Feinstein SC Snider WD Presence or absence of TrkA protein distinguishes subsets of small sensory neurons with unique cytochemical characteristics and dorsal horn projections J Comp Neurol 1995 10 404 416 10.1002/cne.903610305 8550888

[B4] Price TJ Flores CM Critical evaluation of the colocalization between calcitonin gene-related peptide, substance P, transient receptor potential vanilloid subfamily type 1 immunoreactivities, and isolectin B4 binding in primary afferent neurons of the rat and mouse J Pain 2007 10 263 272 17113352 10.1016/j.jpain.2006.09.005 PMC1899162

[B5] Albers KM Woodbury CJ Ritter AM Davis BM Koerber HR Glial cell-line-derived neurotrophic factor expression in skin alters the mechanical sensitivity of cutaneous nociceptors J Neurosci 2006 10 2981 2990 10.1523/JNEUROSCI.4863-05.2006 16540576 PMC6673969

[B6] Bradbury EJ Burnstock G McMahon SB The expression of P2X3 purinoreceptors in sensory neurons: effects of axotomy and glial-derived neurotrophic factor Mol Cell Neurosci 1998 10 256 268 10.1006/mcne.1998.0719 9828090

[B7] Golden JP Hoshi M Nassar MA Enomoto H Wood JN Milbrandt J Gereau RW Johnson EM Jr Jain S RET signaling is required for survival and normal function of nonpeptidergic nociceptors J Neurosci 2010 10 3983 3994 10.1523/JNEUROSCI.5930-09.2010 20237269 PMC2850282

[B8] Molliver DC Wright DE Leitner ML Parsadanian AS Doster K Wen D Yan Q Snider WD IB4-binding DRG neurons switch from NGF to GDNF dependence in early postnatal life Neuron 1997 10 849 861 10.1016/S0896-6273(00)80966-6 9354331

[B9] Silverman JD Kruger L Selective neuronal glycoconjugate expression in sensory and autonomic ganglia: relation of lectin reactivity to peptide and enzyme markers J Neurocytol 1990 10 789 801 10.1007/BF01188046 2077115

[B10] Scherrer G Imamachi N Cao Y-Q Contet C Mennicken F O’Donnell D Kieffer BL Basbaum AI Dissociation of the opioid receptor mechanisms that control mechanical and heat pain Cell 2009 10 1148 1159 10.1016/j.cell.2009.04.019 19524516 PMC3683597

[B11] Braz JM Nassar MA Wood JN Basbaum AI Parallel “pain” pathways arise from subpopulations of primary afferent nociceptor Neuron 2005 10 787 793 10.1016/j.neuron.2005.08.015 16157274

[B12] Cavanaugh DJ Lee H Lo L Shields SD Zylka MJ Basbaum AI Anderson DJ Distinct subsets of unmyelinated primary sensory fibers mediate behavioral responses to noxious thermal and mechanical stimuli Proc Natl Acad Sci 2009 10 9075 9080 10.1073/pnas.0901507106 19451647 PMC2683885

[B13] Dirajlal S Pauers LE Stucky CL Differential response properties of IB(4)-positive and -negative unmyelinated sensory neurons to protons and capsaicin J Neurophysiol 2003 10 513 524 12522198 10.1152/jn.00371.2002

[B14] Stucky CL Lewin GR Isolectin B(4)-positive and -negative nociceptors are functionally distinct J Neurosci 1999 10 6497 6505 10414978 10.1523/JNEUROSCI.19-15-06497.1999 PMC6782829

[B15] Woodbury CJ Zwick M Wang S Lawson JJ Caterina MJ Koltzenburg M Albers KM Koerber HR Davis BM Nociceptors lacking TRPV1 and TRPV2 have normal heat responses J Neurosci 2004 10 6410 6415 10.1523/JNEUROSCI.1421-04.2004 15254097 PMC6729548

[B16] Lawson JJ McIlwrath SL Woodbury CJ Davis BM Koerber HR TRPV1 unlike TRPV2 is restricted to a subset of mechanically insensitive cutaneous nociceptors responding to heat J Pain 2008 10 298 308 10.1016/j.jpain.2007.12.001 18226966 PMC2372162

[B17] Wang HB Zhao B Zhong YQ Li KC Li ZY Wang Q Lu YJ Zhang ZN He SQ Zheng HC Wu SX Hökfelt TG Bao L Zhang X Coexpression of delta- and mu-opioid receptors in nociceptive sensory neurons Proc Natl Acad Sci U S A 2010 10 13117 13122 10.1073/pnas.1008382107 20615975 PMC2919913

[B18] Beaudry H Dubois D Gendron L Activation of spinal mu- and delta-opioid receptors potently inhibits substance P release induced by peripheral noxious stimuli J Neurosci 2011 10 13068 13077 10.1523/JNEUROSCI.1817-11.2011 21917790 PMC3848976

[B19] Snider WD McMahon SB Tackling pain at the source: new ideas about nociceptors Neuron 1998 10 629 632 10.1016/S0896-6273(00)81003-X 9581756

[B20] Mantyh PW Hunt SP Hot peppers and pain Neuron 1998 10 644 645 9808445 10.1016/s0896-6273(00)80575-9

[B21] Ye Y Dang D Zhang J Viet CT Lam DK Dolan JC Gibbs JL Schmidt BL Nerve growth factor links oral cancer progression, pain, and cachexia Mol Cancer Ther 2011 10 1667 1676 10.1158/1535-7163.MCT-11-0123 21750223 PMC3375020

[B22] Ye Y Dang D Viet CT Dolan JC Schmidt BL Analgesia targeting IB4-positive neurons in cancer-induced mechanical hypersensitivity J Pain 2012 10 524 531 10.1016/j.jpain.2012.01.006 22483679 PMC3786360

[B23] Amaya F Shimosato G Nagano M Ueda M Hashimoto S Tanaka Y Suzuki H Tanaka M NGF and GDNF differentially regulate TRPV1 expression that contributes to development of inflammatory thermal hyperalgesia Eur J Neurosci 2004 10 2303 2310 10.1111/j.1460-9568.2004.03701.x 15525272

[B24] Jankowski MP Koerber HR Kruger L, Light AR Neurotrophic factors and nociceptor sensitization Translational Pain Research: From Mouse to Man 2010 Boca Raton, FL: Frontiers in Neuroscience

[B25] Stein AT Ufret-Vincenty CA Hua L Santana LF Gordon SE Phosphoinositide 3-kinase binds to TRPV1 and mediates NGF-stimulated TRPV1 trafficking to the plasma membrane J Gen Physiol 2006 10 509 522 10.1085/jgp.200609576 17074976 PMC2151588

[B26] Zhang X Huang J McNaughton PA NGF rapidly increases membrane expression of TRPV1 heat-gated ion channels EMBO J 2005 10 4211 4223 10.1038/sj.emboj.7600893 16319926 PMC1356334

[B27] Asai H Ozaki N Shinoda M Nagamine K Tohnai I Ueda M Sugiura Y Heat and mechanical hyperalgesia in mice model of cancer pain Pain 2005 10 19 29 10.1016/j.pain.2005.05.010 16043290

[B28] Shinoda M Ogino A Ozaki N Urano H Hironaka K Yasui M Sugiura Y Involvement of TRPV1 in nociceptive behavior in a rat model of cancer pain J Pain 2008 10 687 699 10.1016/j.jpain.2008.02.007 18455478

[B29] Schuller HM Neurotransmission and cancer: implications for prevention and therapy Anticancer Drugs 2008 10 655 671 10.1097/CAD.0b013e3283025b58 18594207

[B30] Mancino M Ametller E Gascon P Almendro V The neuronal influence on tumor progression Biochim Biophys Acta 1816 10 105 118 10.1016/j.bbcan.2011.04.005 21616127

[B31] Fagan JJ Collins B Barnes L D’Amico F Myers EN Johnson JT Perineural invasion in squamous cell carcinoma of the head and neck Arch Otolaryngol Head Neck Surg 1998 10 637 640 10.1001/archotol.124.6.637 9639472

[B32] Kolokythas A Cox DP Dekker N Schmidt BL Nerve growth factor (NGF) and tyrosine kinase A (Trk A) receptor in oral squamous cell carcinoma: is there an association with perineural invasion? J Oral Maxillofac Surg 2010 10 1290 1295 10.1016/j.joms.2010.01.006 20363547

[B33] Rahima B Shingaki S Nagata M Saito C Prognostic significance of perineural invasion in oral and oropharyngeal carcinoma Oral Surg Oral Med Oral Pathol Oral Radiol Endod 2004 10 423 431 10.1016/j.tripleo.2003.10.014 15088027

[B34] Raju B Haug SR Ibrahim SO Heyeraas KJ Sympathectomy decreases size and invasiveness of tongue cancer in rats Neuroscience 2007 10 715 725 10.1016/j.neuroscience.2007.07.048 17916410

[B35] Raju B Hultstrom M Haug SR Ibrahim SO Heyeraas KJ Sympathectomy suppresses tumor growth and alters gene-expression profiles in rat tongue cancer Eur J Oral Sci 2009 10 351 361 10.1111/j.1600-0722.2009.00646.x 19627344

[B36] Feng F Yang J Tong L Yuan S Tian Y Hong L Wang W Zhang H Substance P immunoreactive nerve fibres are related to gastric cancer differentiation status and could promote proliferation and migration of gastric cancer cells Cell Biol Int 2011 10 623 629 10.1042/CBI20100229 21091434

[B37] Vulchanova L Olson TH Stone LS Riedl MS Elde R Honda CN Cytotoxic targeting of isolectin IB4-binding sensory neurons Neuroscience 2001 10 143 155 10.1016/S0306-4522(01)00377-3 11738138

[B38] Nishiguchi J Sasaki K Seki S Chancellor MB Erickson KA de Groat WC Kumon H Yoshimura N Effects of isolectin B4-conjugated saporin, a targeting cytotoxin, on bladder overactivity induced by bladder irritation Eur J Neurosci 2004 10 474 482 10.1111/j.1460-9568.2004.03508.x 15233756

[B39] Joseph EK Chen X Bogen O Levine JD Oxaliplatin acts on IB4-positive nociceptors to induce an oxidative stress-dependent acute painful peripheral neuropathy J Pain 2008 10 463 472 10.1016/j.jpain.2008.01.335 18359667

[B40] Bogen O Joseph EK Chen X Levine JD GDNF hyperalgesia is mediated by PLCgamma, MAPK/ERK, PI3K, CDK5 and Src family kinase signaling and dependent on the IB4-binding protein versican Eur J Neurosci 2008 10 12 19 10.1111/j.1460-9568.2008.06308.x 18616564 PMC2660608

[B41] Zwick M Davis BM Woodbury CJ Burkett JN Koerber HR Simpson JF Albers KM Glial cell line-derived neurotrophic factor is a survival factor for isolectin B4-positive, but not vanilloid receptor 1-positive, neurons in the mouse J Neurosci 2002 10 4057 4065 12019325 10.1523/JNEUROSCI.22-10-04057.2002 PMC6757648

[B42] Fang X Djouhri L McMullan S Berry C Waxman SG Okuse K Lawson SN Intense isolectin-B4 binding in rat dorsal root ganglion neurons distinguishes C-fiber nociceptors with broad action potentials and high Nav1.9 expression J Neurosci 2006 10 7281 7292 10.1523/JNEUROSCI.1072-06.2006 16822986 PMC6673936

[B43] Normandin A Luccarini P Molat JL Gendron L Dallel R Spinal mu and delta opioids inhibit both thermal and mechanical pain in rats J Neurosci 2013 10 11703 11714 10.1523/JNEUROSCI.1631-13.2013 23843537 PMC3855450

[B44] Tarpley JW Kohler MG Martin WJ The behavioral and neuroanatomical effects of IB4-saporin treatment in rat models of nociceptive and neuropathic pain Brain Res 2004 10 65 76 10.1016/j.brainres.2004.09.027 15533317

[B45] Iadarola MJ Mannes AJ The vanilloid agonist resiniferatoxin for interventional-based pain control Curr Top Med Chem 2011 10 2171 2179 10.2174/156802611796904942 21671877 PMC4289604

[B46] Brown DC Iadarola MJ Perkowski SZ Erin H Shofer F Laszlo KJ Olah Z Mannes AJ Physiologic and antinociceptive effects of intrathecal resiniferatoxin in a canine bone cancer model Anesthesiology 2005 10 1052 1059 10.1097/00000542-200511000-00020 16249680

[B47] Joseph EK Levine JD Hyperalgesic priming is restricted to isolectin B4-positive nociceptors Neuroscience 2010 10 431 435 10.1016/j.neuroscience.2010.04.082 20457222 PMC2903040

[B48] Joseph EK Levine JD Mu and delta opioid receptors on nociceptors attenuate mechanical hyperalgesia in rat Neuroscience 2010 10 344 350 10.1016/j.neuroscience.2010.08.035 20736053 PMC2956864

[B49] Hucho TB Dina OA Levine JD Epac mediates a cAMP-to-PKC signaling in inflammatory pain: an isolectin B4(+) neuron-specific mechanism J Neurosci 2005 10 6119 6126 10.1523/JNEUROSCI.0285-05.2005 15987941 PMC6725047

[B50] Connelly ST Schmidt BL Evaluation of pain in patients with oral squamous cell carcinoma J Pain 2004 10 505 510 10.1016/j.jpain.2004.09.002 15556829

[B51] van den Beuken-van Everdingen MH de Rijke JM Kessels AG Schouten HC van Kleef M Patijn J Prevalence of pain in patients with cancer: a systematic review of the past 40 years Ann Oncol 2007 10 1437 1449 10.1093/annonc/mdm056 17355955

[B52] Viet CT Ye Y Dang D Lam DK Achdjian S Zhang J Schmidt BL Re-expression of the methylated EDNRB gene in oral squamous cell carcinoma attenuates cancer-induced pain Pain 2011 10 2323 2332 10.1016/j.pain.2011.06.025 21782343 PMC3375027

[B53] Vulchanova L Riedl MS Shuster SJ Stone LS Hargreaves KM Buell G Surprenant A North RA Elde R P2X3 is expressed by DRG neurons that terminate in inner lamina II Eur J Neurosci 1998 10 3470 3478 10.1046/j.1460-9568.1998.00355.x 9824460

[B54] Barabas ME Kossyreva EA Stucky CL TRPA1 is functionally expressed primarily by IB4-binding, non-peptidergic mouse and rat sensory neurons PLoS One 2012 10 e47988 10.1371/journal.pone.0047988 23133534 PMC3485059

[B55] Taylor AM Osikowicz M Ribeiro-da-Silva A Consequences of the ablation of nonpeptidergic afferents in an animal model of trigeminal neuropathic pain Pain 2012 10 1311 1319 10.1016/j.pain.2012.03.023 22521917

[B56] Pickering V Jay Gupta R Quang P Jordan RC Schmidt BL Effect of peripheral endothelin-1 concentration on carcinoma-induced pain in mice Eur J Pain 2007 10 293 300 17664075 10.1016/j.ejpain.2007.06.001 PMC2771221

[B57] Motta EM Calixto JB Rae GA Mechanical hyperalgesia induced by endothelin-1 in rats is mediated via phospholipase C, protein kinase C, and MAP kinases Exp Biol Med (Maywood) 2006 10 1141 1145 16741065

[B58] Huang J Zhang X McNaughton PA Inflammatory pain: the cellular basis of heat hyperalgesia Curr Neuropharmacol 2006 10 197 206 10.2174/157015906778019554 18615146 PMC2430694

[B59] Kawamata T Ji W Yamamoto J Niiyama Y Furuse S Namiki A Contribution of transient receptor potential vanilloid subfamily 1 to endothelin-1-induced thermal hyperalgesia Neuroscience 2008 10 1067 1076 10.1016/j.neuroscience.2008.04.010 18495351

[B60] Moriyama T Iida T Kobayashi K Higashi T Fukuoka T Tsumura H Leon C Suzuki N Inoue K Gachet C Noguchi K Tominaga M Possible involvement of P2Y2 metabotropic receptors in ATP-induced transient receptor potential vanilloid receptor 1-mediated thermal hypersensitivity J Neurosci 2003 10 6058 6062 12853424 10.1523/JNEUROSCI.23-14-06058.2003 PMC6740351

[B61] Nagamine K Ozaki N Shinoda M Asai H Nishiguchi H Mitsudo K Tohnai I Ueda M Sugiura Y Mechanical allodynia and thermal hyperalgesia induced by experimental squamous cell carcinoma of the lower gingiva in rats J Pain 2006 10 659 670 10.1016/j.jpain.2006.02.013 16942952

[B62] Menendez L Juarez L Garcia E Garcia-Suarez O Hidalgo A Baamonde A Analgesic effects of capsazepine and resiniferatoxin on bone cancer pain in mice Neurosci Lett 2006 10 70 73 10.1016/j.neulet.2005.09.046 16243435

[B63] Menendez L Juarez L Garcia V Hidalgo A Baamonde A Involvement of nitric oxide in the inhibition of bone cancer-induced hyperalgesia through the activation of peripheral opioid receptors in mice Neuropharmacology 2007 10 71 80 10.1016/j.neuropharm.2007.04.011 17543351

[B64] Zinck NDT Downie JW IB4 afferent sprouting contributes to bladder dysfunction in spinal rats Exp Neurol 2008 10 293 302 10.1016/j.expneurol.2008.06.006 18602393

[B65] Dai H Li R Wheeler T Ozen M Ittmann M Anderson M Wang Y Rowley D Younes M Ayala GE Enhanced survival in perineural invasion of pancreatic cancer: an in vitro approach Hum Pathol 2007 10 299 307 10.1016/j.humpath.2006.08.002 17097719

[B66] Li ZJ Cho CH Neurotransmitters, more than meets the eye–neurotransmitters and their perspectives in cancer development and therapy Eur J Pharmacol 2011 10 17 22 10.1016/j.ejphar.2011.05.077 21664902

[B67] Gonzales CB Kirma NB De La Chapa JJ Chen R Henry MA Luo S Hargreaves KM Vanilloids induce oral cancer apoptosis independent of TRPV1 Oral Oncol 2014 [Epud ahead of print] 10.1016/j.oraloncology.2013.12.023 PMC4418484 24434067

